# Introducing an online community into a clinical education setting: a pilot study of student and staff engagement and outcomes using blended learning

**DOI:** 10.1186/1472-6920-10-6

**Published:** 2010-01-26

**Authors:** Kathleen Gray, Jacinta Tobin

**Affiliations:** 1 Faculty of Medicine, Dentistry and Health Sciences, University of Melbourne, Melbourne, Australia

## Abstract

**Background:**

There are growing reasons to use both information and communication functions of learning technologies as part of clinical education, but the literature offers few accounts of such implementations or evaluations of their impact. This paper details the process of implementing a blend of online and face-to-face learning and teaching in a clinical education setting and it reports on the educational impact of this innovation.

**Methods:**

This study designed an online community to complement a series of on-site workshops and monitored its use over a semester. Quantitative and qualitative data recording 43 final-year medical students' and 13 clinical educators' experiences with this blended approach to learning and teaching were analysed using access, adoption and quality criteria as measures of impact.

**Results:**

The introduction of the online community produced high student ratings of the quality of learning and teaching and it produced student academic results that were equivalent to those from face-to-face-only learning and teaching. Staff had mixed views about using blended learning.

**Conclusions:**

Projects such as this take skilled effort and time. Strong incentives are required to encourage clinical staff and students to use a new mode of communication. A more synchronous or multi-channel communication feedback system might stimulate increased adoption. Cultural change in clinical teaching is also required before clinical education can benefit more widely from initiatives such as this.

## Background

The provision of e-learning as part of clinical education is a growing imperative, and is underscored by curriculum accrediting bodies with requirements such as, "Students must be ready to use technology and communications tools as they are used in practice and flexible enough to incorporate changing technology" [1].

E-learning may offer advantages in clinical education, such as overcoming physical limitations of time and space, supporting instructional methods that are hard to achieve using textbooks and reaching a larger number of students without increasing resource requirements. Nevertheless it is not without drawbacks, for instance initial set-up costs, lack of engagement compared to face-to-face interaction and technical problems that can spoil users' overall learning and teaching experiences as Cook has noted [2].

There are few reports of student and staff engagement when face-to-face teaching is blended with e-learning in clinical education, and even fewer reports of educational outcomes from such innovations. Berman, Fall, Maloney and Levine [3] note a "paucity of knowledge regarding how to integrate CAI *[computer-aided instruction] *effectively into clinical education".

The importance of the communication component of e-learning in clinical settings is highlighted by De Wever, Van Winckel and Valcke [4]. E-learning may be most effective in clinical education if it is used not merely to transmit content from teacher to learner but rather to support flexible, engaging and learner-centred teaching, to encourage interaction among students and staff and to enable them to collaborate and communicate asynchronously, according to Ellaway and Masters [5].

Success in integrating e-learning for student-staff communication in clinical settings is dependent on both learners' and teachers' experiences with the innovation. However it cannot be assumed that all medical students are well versed in using information and communication technologies for educational purposes - for example, see findings reported by Kennedy, Gray and Tse about the diversity of technology-based experiences in the student population enrolled in one medical degree [6]. Nor can it be assumed that it is straightforward for clinical educators to make the transition from traditional face-to-face-only to blended online interaction with students as discussed by Lockyer, Sargeant, Curran and Fleet [7].

The purpose of this paper is to add to understanding the use of e-learning in clinical education by:

• providing a detailed account of the process of implementing a blend of online and face-to-face learning and teaching in a clinical education setting; and

• reporting findings from evaluating the impact of this form of e-learning on clinical learning and teaching experiences.

## Methods

The project used a design-based research methodology, an approach "which simultaneously pursues the goals of developing effective learning environments and using such environments as natural laboratories to study learning and teaching ... [and] has evolved primarily as a means for studying innovative learning environments, often including new educational technologies or other complex approaches, in classroom settings" [8]. A precedent for this approach to research into clinical education, in the form of an investigation into another aspect of implementing e-learning, is found in Dornan, Hadfield, Brown, Boshuizen and Scherpbier [9].

The research site was a pilot project undertaken to enhance clinical teaching by setting up an online community (called "Western Workshops Online") that would complement a series of 15 on-site workshops. The pedagogical rationale for these workshops was to strengthen students' readiness to qualify and work as interns by reviewing key topics (such as acid-base balance, adverse drug events, arrhythmia, blood transfusion, cardiopulmonary resuscitation, chest pain, collapse, confusion, death documentation, drug charts, dyspnoea, electrolyte disturbances, emergency, fever and investigation tasks). The workshops were also intended to assist students to build professional relationships with senior clinicians in anticipation of pursuing higher training in an area of interest. Thus one research question was whether a blended approach would enhance, compromise or make no difference to clinical education.

There was also a logistical rationale for the e-learning innovation. Clinical education workshops were problematic to run as a face-to-face-only series. Prior to this project, final year medical students had been offered a program of 15 workshops at a single clinical school site in a base hospital, during their five-week medicine term (thus the program ran three times during a semester). However the clinical school expanded, and each semester half the students were now located at a clinical sub-school site. Travel from the sub-school back to the base hospital could take an hour, and clinical educators at the new sub-school complained about students leaving their placement locations early to attend. A previous effort was made to address the issue by providing real-time access to each workshop in the series from video-conference suites at selected locations, but the disconnect between undergoing day-to-day learning with one set of clinical educators and watching a series of workshops given by a different set of clinical educators contributed to poor student attendance. At the new sub-school, it was not possible to run the program three times because of limited staff availability, so it ran once, over ten weeks. Even so, this posed geographical challenges; while in any given week approximately one-third of students were physically located in the sub-school doing their medicine term, a further third were at the base hospital school doing a surgical term and the remainder were in general practice at varying distances from the main school and sub-school, and with variable and not necessarily compatible real-time communication facilities. Thus another research question was whether staff and students would find a blended approach to be a useful or preferable educational management solution, in comparison to previous approaches.

The pilot project was carried out in the clinical sub-school during the second half of 2008. The project was sponsored by the office of the clinical sub-school dean, resourced by a university fund for learning and teaching improvement, managed by an educational technologist and supported by technical and administrative staff. The authors (the educational technologist and clinical school sub-dean respectively) obtained approval from the University's Human Research Ethics Committee to conduct the research.

Participants in this pilot project were 43 students and 13 clinical educators involved in the final semester (semester 12) of an undergraduate medical degree. (This degree was structured as 5 semesters of university-campus-based education and an intercalated research year followed by 5 semesters of clinical placement rotations, with a small cohort of graduate-entry students doing a truncated preclinical component of 4 semesters and the same clinical time). Most students' use of the medical school's learning management system (LMS) had ended upon their transition from campus to clinical education, three years before this pilot project. The majority of clinical educators had no prior experience with any form of e-learning; all were practising clinicians and none of them spent more than five hours per week doing any form of clinical teaching.

This project began by developing an online community to complement the workshop program. Development occurred in stages, each of which is described in more detail in the following paragraphs. The first four stages were carried out over a 3-month period prior to the workshop series and the final stage ran formally for the 10 weeks of the workshop series:

1. determining aims in relation to the range of clinical education issues;

2. designing online interaction processes and workflows;

3. selecting the most suitable e-learning tools and combinations of tools;

4. advising and training participants about the purposes, processes and tools;

5. supporting and monitoring participants' use of the online community.

The aims of the pilot project were determined in descending order of priority as:

• to make workshop participation more efficient and convenient for students and staff;

• to increase workshop interactions between students and staff and among students and their peers;

• to help students to review and reflect on workshop topics over time;

• to acquaint students and staff with communication technologies and learning methods that might be useful in higher training and continuing professional development.

The design of the online community combined asynchronous discussion forums and media file sharing tools in order to provide week-by-week topic-by-topic access to:

• preparatory clinical scenarios, self-test questions and discussion starters, in the form of a short set of slides, uploaded in the week prior to each on-site workshop;

• an audio-recording of the clinician's presentation and discussion with students during the workshop and accompanying full set of slides, uploaded right after the actual workshop;

• follow-up exchanges of comments, questions and answers, added in the week following the workshop.

The project used the discussion forum feature of the "community" tool in the Blackboard™ system and it used the Lectopia/Echo™ system for collating audio files of lectures and corresponding Powerpoint™ slides. The project opted to use the university-wide LMS - it integrated both systems, it enabled password- and copyright-protected linking to media files from within each weekly topic discussion and it accommodated clinical educator users who were not continuing university staff members. (This combination of features was not found in the medical school LMS previously used by participating students nor in free open source software for online communities).

Participants were inducted into the project in several ways. Clinical educators were contacted personally by the clinical sub-school dean to explain the purpose of the online community, with follow-up emails explaining how to use the system. Students were briefed by the clinical sub-school dean as part of a pre-semester orientation session and received hand-outs and follow-up group emails from the clinical sub-school office. Two incentives were used to build student interest: a small gift voucher was awarded for the best student contribution to each weekly topic discussion, and student contributions were factored into the prize given to the top student at the end of the semester. The 'announcements' tool in the LMS was used to provide reminders to students and staff about the purpose and progress of the community.

Participants' experiences in the online community were captured in the form of quantitative and qualitative data to answer the research questions about the impact of blended learning in terms of effects on learning and user acceptance. The researchers kept written records of their own involvement and elicited the observations of the clinical school administrator who gave routine support to the project. They collected evaluation data from surveys of student and staff participants. They harvested further data from the 'community statistics' tool in the LMS at the end of the semester. They drew upon the final results data for these students and the other half of the clinical school cohort, which was not included in the pilot project, to form a comparative view of students' academic performance.

Analysis of the experiences of students and staff was framed by adapting three criteria from an internationally recognised approach to measuring e-learning impact [10]:

• access - analysing online visits and contributions;

• adoption - analysing observations about user friendliness and feedback about usefulness;

• quality - analysing student marks and student ratings of teaching.

As in studies of similar scale and scope, for example those done by Winckel and Martin [11] and Coughlan and Morar [12], the small scale of this pilot project limited but did not preclude the interpretation of the data collected. Combining the evidence from a variety of data types was used to strengthen the insights derived from this study. Quantitative access data were summarised and described and qualitative access data were analysed using content analysis methods adapted from Salmoni and Gonzalez [13]. Quantitative adoption data were summarised described and qualitative adoption data were analysed thematically. Quantitative data measuring quality were analysed in two ways: the marks of the 43 students participating in the project were statistically compared with those of a cohort of 45 non-participating students in the same semester in the same clinical sub-school; and participating students' ratings of the quality of teaching were analysed descriptively.

## Results and Discussion

### Access: Visits to the online community

Each of the 43 students attended between 3 and 14 on-site workshops in the series (as recorded by clinical school administration staff; records are not available for one workshop). On average there were 25 students in attendance at an on-site workshop. System-generated usage logs show that in comparison, each student made between 0 and 150 visits to the online forum corresponding to each workshop in the series. These logs show a cumulated total of 1528 student visits to the online community over the five month period from July to November with a wide spread of student access by date, day and time. Over the ten-week workshop period, there were twelve dates when the community was visited at least 10 times, fourteen dates when it received at least 20 visits and a further twelve dates with 40 visits or more. In the five-week period between the last workshop and the end-of-year exams there were visits to the community on twenty-five different dates. Saturdays were the only days of the week where there were fewer than 100 visits in all during the semester. The hours between 4 AM and 6 AM were the only times of day when the site was never visited over the semester.

Each of the 13 clinical educators was responsible for one or occasionally two of the workshops in the series. Five of these staff accessed the online community (2, 2, 6, 8 and 23 times), while eight did not access it at all.

It was technically possible for users to access workshop slides and audio-recordings directly from the online file server without going via the online forum (although this was not an intended use of the online community). System-generated statistics show direct accesses to the file server of between 10 and 40 visits per workshop with an average of 23 (but do not differentiate visits by students, teaching staff or other staff).

### Access: Contributions to the online community

Seven students not only visited but also contributed to the online community, between one and five times each, as shown in Table 1. There were 18 contributions mostly of a substantive clinical nature. On average there was just over one student contribution to the online forum for each workshop (it was not possible to analyse how many of the students in attendance at each on-site workshop asked a question or made a comment during the workshop for comparative purposes).

**Table 1:** Student online contributions in relation to content type and workshop attendance

Workshop	1	2	3	4	5	6	7	8	9	10	11	12	13	14	15	
**Student**																**Contributions per student**

A	√	√	√	√	√	√	AQ	√	√	√	SQ√	√		√		2

B	√	√		SC√	AQ+OC√	√		√	√	√	√	√	SC+SQ√	√	√	5

C						√				SC√	AQ	SQ√	√	SC√		4

D	√	√	√	OC√	AQ√	√		√	√		AQ	√			√	3

E	√	√	SQ√	√		√		√	√			√	√	√	√	1

F	√	√	√		√	√		AQ√	√	√	√	√		√		1

G	√	√	√	SQ√	√	√	SQ	√	√	√	√	√		√	√	2

Contributions per workshop	0	0	1	3	3	0	2	1	0	1	3	1	2	1	0	18

Key: Student contributed online content of administrative (A), substantive (S) or other (O) nature in the form of a comment (C) or a question (Q). √ = student attended on-site. Workshop 7 attendance data were not available.

Two of the five staff members who visited the online community made substantive comments in response to student questions and one made but subsequently deleted a contribution. Three other clinical educators arranged for an administrative staff member to post responses to student questions on their behalf.

### Discussion of findings about access

Overall the number of online visits by students is surprisingly large. The wide range of access patterns among students suggests that students who accessed the online community did so at their convenience; given that some students may have opted to download the presentation files rather than re-access them from the file server, the number of accesses per student is not a good indicator of engagement with workshop content, but it shows levels of interest in workshop interaction.

The decline in access at night and at weekends may be explained by some students' lack of internet access outside of the clinical school, and this would have had an impact on the convenience levels experienced by these students. The tail-off in the last four weeks of the series may be explained by students getting closer to their long case and OSCE exams and being more strategic with their time, as well as students being less interested in the content of workshops 11 and 14 because they believed that these were not examinable. The number of student visits both during and after the workshop program suggests that the interaction, revision and reflection aims of the project were accomplished to a large extent.

All seven students who contributed to the online discussion did so in conjunction with attending a lecture; there is only one instance of a student contributing to the online discussion but not attending the lecture. In online discussion such as this, it is desirable to have regular active contribution by many more participants than this, with greater diversity of learning issues, personal perspectives and styles of expression to enliven community interactions. The low but consistent number of online contributions may have been sustained by the incentive offered for the most active contributor each week. The substantive tenor of most of the questions and comments exchanged online between students and staff was appropriate to the workshop topics and project aims.

### Adoption: User friendliness

Some technical issues in the university system were encountered in enrolling non-university-staff as LMS users, resulting in at least four clinical educators experiencing unsuccessful first attempts to log in.

The LMS user interface and communication features were comparatively complicated. For example, to navigate from the LMS main page to view a participant's comment or question about a workshop required 5 click-throughs. To listen to the workshop lecture required between 4 and 8 click-throughs, and initially required some users to download and install media-playing software.

During the actual workshops, technical incompatibility between the lecture hall in the clinical sub-school and the university main campus precluded full use of the Lectopia/Echo™ system's automated audio and slide capture features. Instead, a personal digital audio-recorder with MP3 file recording capability had to be brought to the workshop venue each time (and this device was used with mixed success depending on the operator). Then electronic audio and slide files had to be collated using email or USB data-sticks and manually uploaded.

For students and staff to play their part in each week's schedule of online activities required three closely synchronised steps - preparatory, actual and follow-up. The staff role was dependent on consistent routine support from an administrative staff member to upload files to the LMS and the media file server. Clinical educators were divided as to whether they received adequate support from administrative and technical staff, with one strongly agreeing that they did, four agreeing, one disagreeing, one strongly disagreeing and six not responding to a survey question on this point. Three staff commented on the need to simplify use of the system. A few students commented critically - about delays in making audio files and slides available online (4 comments); staff not supplying answers to student questions online (2); the unfriendliness of the web interface (2); and student lack of easy internet access (1).

### Adoption: Usefulness

Several students made one or more free-text comments about the online community in a standard end-of-semester feedback survey. They were positive about being able to: overcome the difficulty of missing the workshops due to being located at a distance or having other commitments (7 comments); get workshop content in electronic format (4); experience workshop-like interaction (4); revise workshops afterwards (4); and prepare for workshops beforehand (2).

Seven clinical educators gave feedback in an end-of-semester survey in response to the following statements:

"The online initiative was preferable over giving the same lecture on three separate occasions during the semester": 4 agreed; 2 were neutral; 1 one did not respond.

"Providing my lecture preview online seemed to help to engage students in my lecture topic": 2 agreed; 2 were neutral; 2 disagreed; 1 strongly disagreed.

"Providing my recorded lecture online seemed to help to engage students in my lecture topic": 5 were neutral; 1 agreed; 1 one did not respond.

"Looking at student comments/questions posted online gave me insights into how to teach this topic": 3 disagreed; 2 were neutral; 1 agreed; 1 did not respond.

In addition, five clinical educators commented about the online community - there were two comments about the value of interacting with students outside of workshop times, and one comment each about the value of the online community in improving student learning outcomes, the need for more student initiative and the need to change the timing of the on-site workshops.

### Discussion of findings about adoption

Students' open-ended comments were mainly positive and were aligned with the aims of the project regarding convenience, interaction and reflection. Clearly the opportunity to exercise choice and flexibility in learning was welcome. Students' comments about usefulness, combined with their on-site attendance levels, make the point that they did not want to use online methods to replace on-site methods but rather to augment them.

Staff open-ended comments were fewer and more reserved, affirming only the convenience factor. Given that only the lower level of student contributions was visible to staff, but not the much higher level of student visits, staff may have needed better feedback about student activity in the online community in order to form a more positive view of its usefulness.

No definitive patterns of adoption emerged when clinical educators' feedback about the online community was cross-tabulated with their teaching profiles - i.e. with the timing of their workshop in the series, students' rating of their workshop, their total hours of clinical teaching per week and the various types of clinical teaching which they did during the semester. It seemed that clinical educators who were older and more advanced in their career responsibilities were less positive while staff members more junior in their careers and newer to teaching were more enthusiastic. Adoption by some staff may have been weak because the work they had to do to play their part online felt too much like their existing administrative workload of online forms and emails.

Student and staff adoption may have been impeded to some extent by the tools selected, which were not as user-friendly or as flexible as more familiar social web technologies (for instance, Facebook, Flickr or Youtube). The choice of less-than-ideal tools for the online community was partly influenced by some staff concerns about publishing online compared to speaking at workshops on-site. Specifically, staff were apprehensive that clinical advice could be easily replicated or referenced in ways they did not intend or authorise. They were also reluctant to make their workshop presentations available online because they thought that they would lose ownership or control of their teaching materials. Staff were somewhat reassured by the fact that the university system had features (a code of conduct for LMS use, copyright statements on media files and a mechanism to remove student access at the end of the semester) designed to minimise such risks.

### Quality: Student marks

The workshop program was intended in part to improve student performance in each of the three parts of their final year assessment, i.e. tutor assessment in general practice, a long case exam and OSCE stations. It may not be reasonable to assume that the tutor and long case marks represent numerical measures, so non-parametric procedures were used in the analysis of all marks. Table 2 provides the quartiles for each mark by students participating in the project (P) and non-participating students (NP). It suggests strong similarity of the distributions in the two groups.

**Table 2:** Quartiles for each type of mark by student group

	Group	Median	First quartile	Third quartile
Tutor mark	NP	13.0	12.0	13.0

	P	13.0	11.0	14.0

Long case mark	NP	21.0	16.5	21.0

	P	21.0	16.5	21.0

OSCE mark	NP	40.0	36.9	42.3

	P	40.8	37.5	42.9

Total mark	NP	71.7	68.7	76.9

	P	73.4	68.3	78.2

Statistical inferences about the two student groups can be made with the information in Table 3; it provides an estimate of the difference in the location between the two groups with a 95% confidence interval. The associated P-value, reported in the final column, is from the Mann-Whitney test. The difference in location can be interpreted as a difference in the medians (or in any other location parameter). For example, the estimated median difference in the total mark is 0.1; this is a trivial median difference given the scale on which the total score is measured. The 95% confidence interval suggests that observed difference is consistent with true difference in location of the total mark between -2.6 and 2.7; median differences of these magnitudes for the total mark are also relatively small.

**Table 3:** Estimates of the difference in locations between the marks of two student groups

	Difference in locations (NP - P)		
	**Estimate**	**95% confidence interval**	**P-value**

Tutor mark	0.0	-1.0, 1.0	0.5

Long case mark	0.0	-0.1, 4.5	0.3

OSCE mark	-0.5	-1.9, 1.0	0.5

Total mark	0.1	-2.6, 2.7	0.9

Plotting participants' final marks against the number of visits they made to the online community had no predictive value. There were three outliers who each logged on over 100 times and whose final results were in the bottom half of the class. Further research would be required to determine whether this reflected other student learning issues, for example, whether the number of visits to the online community was an expression of anxiety or isolation.

### Quality: Student ratings of quality of teaching

Thirty-seven of the participating medical students rated the helpfulness of the workshop program to their final semester of clinical education in an end-of-semester feedback survey. The survey used a 5-point scale (1 = rarely helpful; 2 = not very often helpful; 3 = on average helpful; 4 = usually helpful; 5 = very helpful). Students rated the workshop program overall at 4.0; they rated the individual on-site workshops between 3.1 and 4.4 with the average individual workshop rating 3.8. They gave the online community specifically an average rating of 3.8 - eight students rated it 5, sixteen students rated it 4, ten students rated it 3 and three students rated it 2.

### Discussion of findings about quality

Students' final marks show that using a blended mode of learning and teaching produced assessment outcomes that were indistinguishable from those achieved using face-to-face-only methods.

Longer term research would be needed to determine effects on student marks from using blended learning over a longer clinical education period, or to determine whether the project was able to deliver other intended learning outcomes (such as learning to use communication technologies for clinical practice and continuing education).

The online community achieved quality-of-teaching ratings that were positive by clinical school standards. Clinical students' appraisals of their experiences are notoriously critical, affected by various factors such as the emotionally-charged environment of the hospital, evaluation fatigue from multiple sampling throughout the medical degree and the perception of gaining no personal benefit from investing effort in improving learning and teaching for subsequent student cohorts.

An unanticipated quality outcome associated with this project was that one of the teachers in the program who was a comparatively conscientious user of the online community was one of those most highly rated overall by students and went on to become 'teacher of the year' for the whole clinical school. The use of the online community as an alternative to the previous practice of presenting the same workshop three times in a semester might be expected to mitigate teacher fatigue and its deleterious effects on the quality of teaching. However comparative studies of this were not possible in this project.

The project provided a continuing professional development opportunity for clinical educators based on action learning. Its potential impacts on their other clinical teaching, on their longer term interest in using technologies for learning and teaching or on their influence on peers' teaching practices cannot be determined at this time.

## Conclusions

This pilot project aimed to blend online activity with face-to-face workshops to improve engagement and outcomes for students and staff, in the face of logistical challenges to effective clinical education and in the absence of exemplars to guide e-learning implementations.

Evaluation of this innovation's impact found that many students accessed the Western Workshops Online community frequently although most were not yet active contributors to discussion, and that staff activity online was limited and was dependent in part on administrative support. Despite limitations in the design of the system, students appeared to accept this way of enhancing their clinical learning, however staff did not appear to be aware of students' acceptance and from their perspective it had very limited utility. The introduction of the online community produced high student ratings of the quality of learning and teaching and it produced student academic results that were equivalent to those from face-to-face-only learning and teaching.

The student learning outcomes reported here from blending online and on-site learning are important for medical schools in which clinical learning is location-dependent and logistically complex and in which clinical teaching is constrained by competing claims on specialists' time. Clinical education that uses blended learning for communication and reflection may provide flexibility to address clinical placement logistics and may enhance student learning. But several issues that arose in this project must be addressed to provide the basis for a successful online clinical education community, and there are four key things for educators to keep in mind:

1. Neither clinical students nor staff will make the change to becoming active in an online community without strong motivations.

2. The investment in working out how to manage an online community is substantial and may be recouped only after several cycles of innovation and improvement.

3. An online community may augment interpersonal communication in clinical settings but does not equate to the stimulus of face-to-face interaction.

4. In order for students to regard using an online community as professionally valuable they must see senior staff modelling a collective approach to its use.

In building community, we should not underestimate the degree of support required to enable busy clinicians to use non-intuitive online interfaces. Asking clinical educators to take an interest in a stand-alone LMS that is unrelated to standard communication systems in their hospitals or private medical practices may be more of an impost than asking them to repeat the same presentation in person. We should also recognise the degree of motivation that busy students need to take full advantage of learning enhancements. Offering online activities more closely aligned with assessment, for example designing the online community to provide more long case practice or OSCE practice, might have increased active contributions by students.

Projects such as this pilot take skilled effort and time and are not likely to be done well by amateurs or for free, but if the work is done systematically the recurrent use of the online community can minimise risks and recoup development costs over a reasonably short time span. Its operation at a much larger scale is possible without much further investment in development, and might well produce a more dynamic community and sustainable resource. However it is important not to underestimate the additional planning and commitment needed if further sophistication in using technology to support clinical education is desired (for example, online simulation or assessment). Broad engagement of clinical students and staff in blended on-site and online learning may also require improvements in the administrative and computing support available to them in clinical settings.

Delays in real-time information and feedback about what is going on in any community slow down the sense of immediacy of involvement and such delays need to be addressed in further iterations of this project. Staff might be more responsive to a more synchronous or multi-channel system for communicating with students - or at least for getting information about student activity - using social web options such as internet chat, email alerting, syndication (RSS), micro-blogging or web-to-mobile phone messaging, for example. Students too might be more inclined to be active participants if they received more frequent stimuli from teachers and from peers.

However technological facilities alone are an insufficient response to the imperative for students to become practised in clinical information and communication technology skills, since clinical students are learning "not only clinical skills but also how to be health professionals" as Egan and Jaye have said [14]. Nor will generational change necessarily resolve the impasse. The hidden curriculum or tacit expectation in clinical education, namely that students will learn to adopt the professional attitudes and behaviours of senior clinicians in a range of situations, has a lasting influence on students' own beliefs and practices, and clinical teaching is one such situation. Senior clinicians must become more positive about learning and teaching with technology before clinical education can benefit more widely from initiatives such as that reported in this project. Strong leadership within the medical profession is required for this kind of cultural change in clinical teaching.

## Competing interests

The authors declare that they have no competing interests.

## Authors' contributions

JT conceived of the pilot study, sponsored its implementation and analysed the quantitative data. KG designed the pilot study, managed its implementation, analysed the qualitative data and drafted and revised the manuscript. Both authors read and approved the final manuscript.

## Authors' information

Kathleen Gray is a Senior Research Fellow in Health Informatics; she holds a PhD in online learning.

Jacinta Tobin, MBBS, PhD, is a Clinical School Sub-Dean and paediatric gastroenterologist.

## Pre-publication history

The pre-publication history for this paper can be accessed here:

http://www.biomedcentral.com/1472-6920/10/6/prepub
